# Youth Care Stories: A Scoping Review Into What, Why and How Narrative Approaches Understand and Improve Quality of Care for Young People

**DOI:** 10.1111/hex.70166

**Published:** 2025-03-14

**Authors:** Eline Verheijen, Suzanne Rutz, Charlotte Barendregt, Anne Margriet Pot

**Affiliations:** ^1^ Institute of Management Research Radboud University Nijmegen Nijmegen the Netherlands; ^2^ Lectorate of Youth Care in Transformation The Hague University of Applied Sciences The Hague the Netherlands; ^3^ Dutch Health and Youth Care Inspectorate Utrecht the Netherlands; ^4^ Erasmus School of Health Policy and Management Erasmus University Rotterdam Rotterdam the Netherlands; ^5^ Optentia, North‐West University Vanderbijlpark South Africa

**Keywords:** creative methods, narrative approaches, quality of care, stories, youth care

## Abstract

**Introduction:**

Narrative approaches are increasingly used to capture the experiences of young people and parents with care services. These approaches are thought to be inclusive and participatory, opening up new perspectives on care quality and its improvement. This scoping review explores the use, rationale, benefits, and challenges of narrative approaches in understanding the quality of care for young people and their families.

**Methods:**

A scoping review was conducted, searching PsycINFO, Web of Science, Scopus, Pubmed, Social Services Abstracts and International Bibliography of the Social Sciences from inception to March 2022. The review targeted studies employing narrative approaches, particularly creative and participative methods, to explore the quality of care for youngsters and parents. Data from 28 studies were extracted, coded, and thematically analysed.

**Results:**

Various narrative approaches were identified, employing diverse forms of expression and participation. These approaches aimed to reveal new insights into care quality from the perspectives of youth and their parents, in addition to dominant professional views. Moreover, narrative approaches are utilised to foster participant reflection, for empowerment and the creation of customised services. Nevertheless, there are persistent concerns about representation, power dynamics and the potential for effecting change.

**Conclusion:**

Narrative approaches contribute to a comprehensive understanding of care quality. However, reflection and further research are required to explore how narrative approaches foster inclusivity, participation, and improvement of care.

**Patient and Public Contribution:**

Members of client/patient organisations were part of the research consortium and contributed to the review setup and the interpretation of findings.

## 1 Introduction

Narrative approaches are increasingly used to collect the personal stories of children and their parents or caregivers on their experiences with youth care services [1, 2, 3]. Specifically, users are currently seen as a rich source of data to identify new aspects of quality of care, in addition to professional and expert knowledge, and hence accommodate a thorough and nuanced understanding of the experienced care quality [4, 5]. Narrative approaches focus on gathering stories, as they offer valuable insights into people's perspectives, experiences, needs and values [6, 7]. Narrative approaches are particularly characterised as inclusive, accessible for a diverse public and a way to include vulnerable target audiences that are often marginalised and excluded [8, 9, 10]. In addition, narrative approaches are characterised as participatory, acknowledging that children and families with care experiences are often best placed to co‐produce insights on how care services will have a positive impact on their lives. Hence participation is increasingly seen as essential to innovate care services and realise care quality improvement [5, 8, 10]. Consequently, narratives are viewed as a powerful approach to put the viewpoints of often excluded users in a vulnerable position at the forefront in quality enhancement. This growing interest extends beyond academia to care organisations striving to improve service quality and to regulators, exploring alternative approaches to assess and involve users in inspections, fostering a more reflexive regulatory framework [11, 12]. Although we know that various narrative approaches are used in the context of care for youngsters, to our knowledge so far oversight is lacking on the methods used, central premises and their benefits and challenges. This scoping review aims to explore their utilisation, using the following research questions:

1. What narrative approaches are used to understand care quality for young people and parents?
2. What are the rationales to use narrative approaches?
3. What benefits and challenges accompany their utilisation?

By addressing these, this review seeks to shed light on the landscape of narrative approaches in the field of youth care, offering insights for their integration into quality improvement initiatives and regulatory practices.

Despite the diverse caregiving arrangements in youth care, the term “parents” is used here for readability, encompassing all caregivers, including adoptive parents and family members.

## 2 Methods

To identify narrative approaches used for youth services, we conducted a scoping review, with a strict search strategy, following Arksey and O'Malley's recommendations [13].

### 2.1 Search Strategies

For this scoping review, with the assistance of an information specialist, a comprehensive search was performed in the bibliographic databases PsycINFO, Web of Science, Scopus, Pubmed, Social Services Abstracts and International Bibliography of the Social Sciences from inception to March 2022. Search terms included controlled terms as well as free text terms. As we aimed to gain a broad overview of narrative approaches, we focused on care for children in a broad sense, including for instance social and welfare services, mental health and medical care to identify the research activities in this field [13]. Search terms expressing (variants for) ‘narrative approach’ were combined with search terms comprising (variants for) ‘care quality’ and (variants for) ‘age’ (see Table 1)^1^. These concepts were searched in the title, keywords and abstract. The search was limited to publications in English based on the advice of the information specialist. Following completion of the search, duplicates were removed using RefWorks.

**Table 1:** Search terms used.

Sector	Narrative approach	Quality of services	Division
Social service	Narrative	Regulation/regulator	Child
Child welfare	Storytelling	Inspection/inspector	Youth
Mental health service	Patient Story	Accountability/accountable	Teen
Mental disability	Patient perspective	Justification	Juvenile
Youth mental care	Discourse	Assessment	Adolescent
Social casework	Patient attitude	Quality	
Social work	Client attitude	Policy	
Foster home	Client participation	Policies	
Child protection	Patient participation	Health service	
Child service	Client engagement	Health care	
Youth care	Patient engagement	Medical service	
Youth custody		Medical care	
Youth work			

### 2.2 Study Selection and Data Extraction

Two researchers (RS and BC) independently screened the records' title and abstract for potentially relevant studies using Rayyan, a program that structures scoping reviews. Initially, records were included if they: (1) used narrative approaches; (2) focused on care quality for youngsters and parents; and (3) were qualitative empirical studies. Records were excluded if: (1) the perspective was not that of a youngster or parent (e.g., papers on professionals' perspectives were excluded); and (2) focus was on adults reminiscing about their childhood, as our focus was on narrative approaches that gain insight in current care quality. Discrepancies between researchers RS and BC were resolved by a third researcher (VE). To ensure the inter‐coder reliability, meetings were held after screening 5%, 50% and 100% of the records to address uncertainties and discrepancies.

In the second stage, records were assessed for eligibility by reading the full text. Records were included if the narrative approaches entailed: (1) participatory aspects; or (2) a creative component, both aiming to encompass diverse groups of youth, including those with difficulties to verbally express themselves. Records based solely on semi‐structured interviews were excluded. Although semi‐structured interviews may be useful to comprehend care quality, they limit the narrative element—the collection of people's own stories—and the participation of young people and parents. Narrative methods allow storytellers to interpret and reflect on their own experiences, needs and values [6], unlike semi‐structured interviews that steer questions by using a format and often leave interpretation of responses to researchers. Records with semi‐structured interviews were only incorporated if they complemented individuals' stories, that were retrieved by creative methods, such as digital storytelling or PhotoVoice.

**Figure 1:**
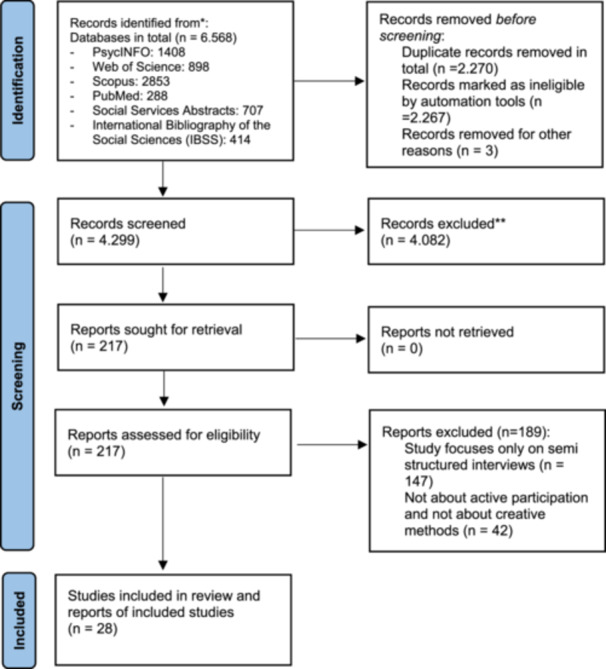
PRISMA diagram.

SR and VE each assessed 50% of the records. They met to discuss uncertainties and to decide for the record's in‐ or exclusion. Figure 1 shows the process of record identification, screening, selection, and review, following guidelines from prisma‐statement.org [14].

VE and RS developed a data extraction sheet to capture key elements of the included studies. This sheet recorded details such as publication year, subject, sector, focus on parents or young people, age of the young people (target audience), a summary, and the narrative approaches employed (verbal/non‐verbal aspects, mode of engagement, etc.). Initially, RS and VE each extracted publication year, subject, target audience, and a summary for 50% of the studies. Subsequently, they extracted narrative approach data for the remaining studies. Both researchers then reviewed all included studies, cross‐checked for inconsistencies, and supplemented extracted data.

### 2.3 Synthesis of Results

The researchers used the data from extraction sheet to analyse (1) distinct narrative approaches in services for youth and families; (2) reasons for their utilisation; and (3) associated benefits and challenges.

### 2.4 Consultation

This review was executed within the RUN consortium, where client/patient organisations, regulators and researchers cooperate to develop reflexive health care regulation using narrative approaches. We presented our review setup and initial findings at two consortium meetings in November 2022 and April 2023. Feedback stressed the need for inclusivity, especially for young people and parents who struggle to articulate verbally. Consequently, we specifically included narrative methods with creative components and critically assessed participation‐related aspects.

## 3 Findings

Twenty‐eight studies that met the inclusion criteria were found eligible for this review, spanning publication years from 1998 to 2022 and originating from nine countries, with a predominant focus in the United Kingdom (*n* = 8), Canada (*n* = 7) and the United States (*n* = 6). In our study, we differentiate between children aged 12 and under and adolescents over the age of 12, since the age of 12 marks significant differences in education, healthcare, legal contexts and developmental stages in modern societies [15]. The majority of studies focused on adolescents (*n* = 18), with a smaller portion involving children (*n* = 1) or parents (*n* = 3), or combinations of children and adolescents (*n* = 3), children, adolescents and parents (*n* = 2) or children and parents (*n* = 1). Domains covered included youth care (*n* = 10), medical care (*n* = 5), mental care (*n* = 5), mental care and education (*n* = 4), social services (*n* = 3), and medical care and social services (*n* = 1). Further details are provided in Table 2. Since we conducted a search into academic literature, many narrative approaches focus on narrative research methods.

**Table 2:** Characteristics of the narrative approaches used (*N* = 28).

First author(s)	Description	Healthcare sector	Target audience	Narrative approaches(s)
Brion‐Meisels [16]	Youth researchers conduct a study including surveys, interviews, visual methods, ethnographic methods, and a focus group about mental wellbeing and seeking support.	Mental health, education	Adolescents	Participatory action research, interviews, focus groups, survey
Brown and Dixon [17]	Young peoples' photo representations of mental health were collected and used to stimulate focus group discussions with students across 7 schools.	Mental health, education	Adolescents	Photo elicited focus groups
Capous‐Desyllas and Mountz [18]	Youth with an LGBTQ background were interviewed to collect their pathways into foster care. PhotoVoice methodology invited youth to visually represent their experiences before, during, and after foster care.	Youth care	Adolescents	PhotoVoice, interviews
Chumbler et al. [19]	Pregnant and parenting adolescents used a journaling tool to express their thoughts in a real‐time, reflexive manner. The journals were sectioned in 5 categories: quizzes, writing prompts, drawing activities, guided journaling and free journaling.	Medical care, social services	Parents	Diary study
Coad et al. [20]	A youth council was set up to contribute to the hospital's policy and services. The process and impact of this youth council was evaluated.	Medical care	Adolescents	Youth advisory group
Collings et al. [21]	Mothers whose children have been placed out of home shared their experiences by mapping their body and using images to represent their experiences, feelings and ideas.	Youth care	Parents	Body mapping
Cossar et al. [22]	Children are interviewed about the extent to which they understood and participated in the child protection process. Interviews were conducted during play activities and supplemented by themed cards.	Youth care	Children; adolescents	Activity‐based interviews
Doucet et al. [23]	Young people participate in a PhotoVoice project, acting as co‐researchers, to share their experiences regarding their relationship with their youth worker.	Youth care	Adolescents	Participatory action research (PAR), PhotoVoice
Elliot‐Groves [24]	Cowichan tribe youth shared their mental wellbeing experiences through nature walks, art workshops, and narrative interviews. A focus group with elders ensured alignment with tribal knowledge.	Mental health	Adolescents	Nature walk, art workshops, interviews
Engqvist et al. [25]	The researchers selected and analysed internet blogs of mothers with postpartum psychotic episodes.	Medical care	Parents	Blog analysis
Fraser et al. [26]	Young Inuit people create videos sharing their experiences with residential care and their insights on good care quality in their youth care units.	Youth care	Adolescents	Video production, interviews, participant observations
Greco et al. [27]	Children shared their experiences of mental support in the school setting, creating life books from photos and images. The project had a participatory approach, with children as co‐researchers.	Mental health, education	Children	PhotoVoice, interviews, participant observations, group sessions
Hall et al. [28]	Young mental health services users created short videos about the concept of positive health, using images, voice recording, music or text. The participants were trained to use the method and were also interviewed.	Mental health	Adolescents	Digital storytelling (via video's, photo's, music, etc.), interviews
Heron and Steckley [29]	Young people shared their experiences on the decisions made with and about them by social services, using digital storytelling. Using videos and music, they and parents co‐created a single story featuring a fictional character.	Social services	Adolescents	Digital storytelling (via video's, music, etc.), interviews, focus group
Hudson et al. [30]	Homeless drug‐using young adults participate in a community advisory board which aims to gain insight in their healthcare seeking behaviour and improve services for homeless.	Social services	Adolescents	Participatory action research, interviews, focus groups
Lal et al. [31]	Youth mental health service users diagnosed with psychosis were interviewed and invited for photography elicited focus groups to gain insight in what they perceived as mental wellbeing.	Mental health	Adolescents	Interviews, photo elicited focus groups
Liabo et al. [32]	Young people who aged out of foster or residential care shared their experiences through interviews and participatory meetings by reflection and creating collages.	Youth care	Adolescents	Interviews, workshops, collages
Luz et al. [33]	Young people discuss their health concepts and needs in reflective workshops, using visuals like magazines and pictures to illustrate their discussions.	Mental health, education	Adolescents	Reflection workshops
Machenjedze et al. [34]	Young people with HIV‐care, living in youth care centres were asked to make drawings of what enabled them to cope with their lives and to write short supporting narratives.	Youth care	Adolescents	Draw‐and‐write techniques
Mackworth‐Young et al. [35]	In participatory workshops and interviews, young women shared their experiences with dealing with HIV Concept mapping, collages and vignettes were used in the workshops.	Medical care	Adolescents	Interviews, workshops (with concept mapping, vignettes and collages)
Marcus et al. [36]	Internet blogs of young people on mental health were selected and analysed.	Mental health	Adolescents	Blog analyses
Martin [37]	Young people were invited to share their transition experiences from youth care to adult care. The researcher then co‐wrote and analysed their stories with the participants.	Youth care	Adolescents	Storytelling
Noyes [38]	Young peopple with chronic lung diseases using ventilators and their family members were interviewed. Given speaking difficulties, researchers employed additional techniques including taking pictures and talk/play and draw/play methods.	Medical care	Children; adolescents; parents	Interviews with additional techniques (pictures, draw and play)
Percy‐Smith and Dalrymple [39]	Children and parents and researchers co‐produce a ‘river of experience’ to map events, experiences and needs from their birth up until now, in order to understand their care journeys and to inform how services might respond better.	Youth care	Children; adolescents; parents	Visual journey mapping, interviews
Pölkki et al. [40]	Written narratives were collected in a writing competition on ‘how my live changed after my family member became mentally ill’. Interviews were held on the same theme.	Mental health	Children; adolescents	Written stories, interviews
Rich et al. [41].	Young peopledocumented their experiences on living with asthma by recording video's on their daily lives, interviews with families and friends, and their reflections on the disease.	Medical care	Children; adolescents	Visual narratives through Video Intervention/Prevention Assessment (VIA)
Richards [42]	Experiences with adoption were gathered through semi‐structured interviews with parents. Children engaged in a creative journal activity, expressing themselves through storytelling, drawings, photos, and small artifacts.	Youth care	Children; parents	Journal, interviews
Schweitzer [43]	Homeless young people participated in a youth council to develop a program for help and assistance. The youngsters collected and analysed data from focus groups.	Social services	Adolescents	Participatory action research (youth advisory group, focus groups)

### 3.1 RQ1: Which Narrative Approaches Are Used?

First, we first looked at how the narrative approaches enabled young people and parents to express themselves. We observed a broad range of expression methods used in narrative approaches, including drawing, photography, videography, digital art, collages, mixed media, blogging, journaling, fiction and non‐fiction writing. These narrative approaches focus on including diverse target audiences. Subsequently, as narrative approaches are thought to be inclusive and participatory [8, 10, 11], we focused on two related characteristics; (1) verbal and nonverbal aspects, with non‐verbal methods being more inclusive for young children and young people with limited verbal expression or literacy; and (2) modes of engagement as this impacts youth participation in narrative approaches.

#### 3.1.1 Verbal and Non‐Verbal Aspects

Verbal approaches [20, 30, 33, 37, 43] focused on oral methods, for example, focus groups, interviews, youth councils. Nonverbal methods were approaches with a focus on nonspoken, for example, written words such as blogs and diaries, drawings, photos, collages, body mapping, music [19, 25, 34, 36, 41]. Authors often utilised a combination of approaches to ensure inclusivity, as verbal methods may pose challenges for some individuals [16, 17, 18, 21, 22, 23, 24, 26, 27, 28, 29, 31, 32, 35, 38, 39, 40, 42]. For instance, Noyes [38] interviewed youngsters with chronic lung diseases who had difficulty speaking, prompting the use of additional nonverbal techniques like photography and draw‐and‐play methods.

#### 3.1.2 Participation in Narrative Approaches

Two studies did not incorporate participant involvement in their approach, using publicly available blogs from the internet instead [25, 36]. In various narrative approaches participation was limited to taking part in interviews or focus groups with activities [17, 26, 27, 28, 32, 35, 41], attending a workshop [32, 33, 35], writing a story [40], keeping a journal or diary [19, 42], or making a drawing [34]. Other approaches involved deeper participation, such as mapping and analysing the journey through organisations with parents and children [39], visualising and analysing the influence of child removal with parents [21], writing and analysing a story on the transition from youth to adult care [37], and using audiovisual methods like PhotoVoice [17, 18, 27], video productions [26, 41] or digital story telling [28, 29].

Participation could also entail developing the narrative approach together with children or youth [20, 22, 24], which was the most tangible in participatory action research [16, 23, 30, 43] and in the narrative projects that had young people as part of the team [18].

### 3.2 RQ2: Rationales for Using Narrative Approaches

In the studies we found, researchers describe four compelling reasons to use narrative approaches.

#### 3.2.1 New Perspectives on Quality of Care

First, narrative approaches are used to provide a new perspective on quality of care [16, 25, 28, 30, 31, 33, 36, 38, 40, 41, 42]. These approaches put the perspective of young people and families centre stage to gain a deeper understanding of their view on care quality. Authors state that they want to listen to young people whose voices otherwise stay unheard. Elliott‐Groves [24] for instance describes how co‐production with indigenous young people was chosen to gain understanding of important tribal concepts to design cultural sensitive healthcare. Authors also use narratives methods to open up new ways of knowing, that counter dominant professional views. For instance, Rich et al. [41] aimed to move beyond the biomedical model of asthma through a video production with youth. In addition, visual narrative methods are selected to activate senses as touch and view to enable access to new ways of knowing.

#### 3.2.2 Reflection on Experiences With Quality of Care

Second, narrative approaches are used to offer opportunities for reflection, allowing youth and families to explain and contemplate on their experiences [19, 21, 23, 29, 37]. Chumbler et al. [19] for instance use a journaling tool to: *“provide insight into the thoughts, concerns and attitudes of adolescent mothers on pregnancy and parenting, allowing for private and immediate reflection during the time of stressful events”*.

#### 3.2.3 Empowerment and Ownership to Reach Inclusion

Third, authors describe that they employ narrative approaches to empower young people and their families by giving them a platform ‘for expression' [18, 23, 24, 27, 32, 37, 43]. Capous‐Desyllas and Mountz [18] enabled youth with an LGBT‐background to share their experiences with foster care through PhotoVoice, granting them agency over what they find important regarding foster care and to empower this marginalised group. Authors argue that the participatory component of narrative approaches, such as PhotoVoice and youth councils, supports youngsters and parents to develop new skills, advocate for themselves and achieve self‐determination.

#### 3.2.4 Tailored Services and Quality Improvement

Lastly, narrative approaches are used to design services with better quality and policies with greater impact, as these become tailored to the needs of youth and families [20, 29, 34]. In doing so, these narrative approaches focus on mobilising change within care services that is based on the perspectives of youth and families. Heron and Steckley [29] for instance aimed to redesign an instrument for collecting young people's views before key decisions on their situation. The existing tool had low usage, prompting the need for enhancement.

### 3.3 RQ3: Benefits and Challenges of Narrative Approaches

In 25 of the 28 studies, authors reflected on benefits and challenges of the narrative approaches. These are intrinsically related to the rationales for using narrative methods.

#### 3.3.1 New Perspectives and Representations

Above we described that authors use narrative approaches to gain a deep understanding of the views of young people and families. Authors describe how the narrative approaches indeed strengthen the participants' understanding of their own situation and increases understanding for researchers and practitioners, offering new and innovative views [17, 18, 20, 21, 25, 26, 28, 29, 36, 38, 40]. Collings et al. [21] for instance describe how body mapping creates ‘a portal for otherwise unsayable feelings, novel ideals and unexplored connections to arise’. Authors also mention that young people and families offer a broader and more holistic view than professionals. Engqvist et al. [25] for instance states that: ‘Professionals often only see a small part of the disorder, while the women [mothers that blogged on postpartum psychosis] describe the whole’*.*

The authors describe representation as an issue that limits these potential new views. Authors mention that various narrative approaches attract young people and parents that have specific skills or are comfortable with the approach. Many narrative approaches for instance rely on verbal skills, which asks that people are able to reflect on their experiences, formulate their vision and speak this out loud. Consequently, the views of those lacking these skills are excluded. At the same time authors also describe various approaches as fun, matching with children's lives and development.

#### 3.3.2 Reflection on Experiences

Authors show that various narrative approaches aid participants to process their experiences [18, 21, 23, 25, 26, 27, 29, 35, 36, 37, 38, 39]. They argue that these approaches that stimulate reflection have a positive therapeutic function and describe this as a strength, specifically when the approach includes joined reflection with peers [23, 27]. Yet, this process may not be so easy and as a consequence may also be intense for participants and for researchers [23].

#### 3.3.3 Empowerment, Ownership and Power Balance

According to studies found, the promise of narrative approaches to empower youth and families comes with various benefits and challenges. Authors describe how participants gained confidence and boosted their self‐esteem as they realised that their opinion mattered. Various papers emphasise the cooperative and co‐creation aspects and the agency coming with it as a strength [18, 19, 20, 24, 27, 29, 32, 33, 37, 42, 43]. Elliott‐Groves [24] for instance describes how the narrative approach disrupted traditional power dynamics between youngsters and professionals.

Yet, various papers show that this powershift is not automatic and can also pose challenges. In a PhotoVoice project, for instance, authors omitted photos with illegal substances to navigate ethical dilemmas, but as a consequence overruled the young people involved. Authors also describe how they take the lead to act on the children's interests and to make sure they are involved meaningfully, which limits youngsters' own involvement [20].

#### 3.3.4 Tailored Services and Quality Improvement

Authors describe how narrative approaches not only enhance tailored services but also foster broader change. They describe as a strength that narrative approaches facilitate network and community building, especially when the narrative approach has a participatory aspect [20, 21, 23, 27, 29, 34, 40]. The approaches then connect people with shared experiences with others, such as community leaders. Moreover, in various papers, it is argued that the other ways of knowing that narrative approaches open up may enhance change. Collings et al. [21] for instance argue that the images on the body maps of mothers whose children have been placed out of home reflect sensitive feelings and symbols that words cannot do. And in PhotoVoice projects, authors state that the visuals help to disseminate the new insights and allow for the findings to translate to social action and improvement [27]. However, they do not show that the approaches have actually led to change.

In addition, various authors show that improving services also present challenges. Professionals and policymakers must acknowledge that their knowledge is incomplete and be able consider new views and take action [29]. Coad et al. [20] for instance show that members of a youth council were only involved in small matters, such as furnishing the hospital ward. Bigger issues concerning tailored services remained underexposed.

## 4 Discussion

In this scoping review, we found a broad variety of narrative approaches to gain insight into the quality of care from the perspective of young people and parents. Rationales to use narrative approaches are to include groups of youngsters and parents that are often excluded in care quality evaluation. Consequently, these methods are assumed to unravel new aspects of care quality. This is important, since quality of care is a complex multifaceted concept, with different meanings for different stakeholders. Narrative approaches may broaden our understanding of care quality which is traditionally based on a professional and medical perspective [2, 3]. Other rationales for using narrative approaches are that they facilitate reflection of participant experiences, empower participants, help design tailored services and realise quality improvement. However, despite the aim to be inclusive, participative and improve quality, there are concerns about representation, power dynamics and impact. From our point of view, three points deserve further consideration.

The first entails the importance of inclusivity. Although narrative approaches are thought to be accessible to diverse groups, the studies also describe that participants require specific skills to join, such as verbal capacities. Many of the narrative approaches we found—even creative approaches—rely partly on verbal skills, which not everyone possesses [8]. It is important when using such approaches, to carefully consider what they ask of participants and also who they may potentially exclude. Researchers should place greater emphasis on the needs and perspectives of their target audience, connecting with young people's experiences and every day lives. For example, the PhotoVoice method allows young people to express themselves through images rather than language, aligning with their digital lives [18]. Building on this, it is important that researchers adapt to the needs of young people, rather than expecting them to fit researchers' methods.

The second point is about power shifts. Various narrative approaches strive to involve participants throughout the process, to open up new knowledge and to encourage change. Yet, our review also finds associated difficulties and ethical dilemmas. Researchers wield significant influence over participants' involvement and input, for instance by excluding photos brought forward by young people that depict illegal practices [18]. For researchers, policymakers, professionals and regulators, it is important to reflect on how the narrative approaches steer the participants' involvement and input. Moreover, in successful participation participants have power to influence the process. This may be challenging for researchers, professionals and the policymakers involved [5], and is especially challenging for regulators, given their hierarchical structures and the predetermined inspection processes [44].

The third point focuses on realising quality improvement. It is assumed that young peoples' narratives can inspire others to make necessary improvements in care, especially through creative expressions like photos and body maps. However, we noticed that the studies do not describe whether this has actually occurred, leaving it as a hypothesis subject to further research. And gaining insights into youth and parents' perspectives is believed to target specific improvements that are meaningful to youngsters and parents. However, our review suggest that this doesn't always result in significant changes. This is in line with previous research into participation and quality improvement, which also show limited results and difficulties to implement necessary changes from the views of care users [44]. Therefore, it is important to not only strive for major improvements in care quality but to also recognise the potential for smaller, nuanced changes, such as building networks with youth and professionals in which they share views.

This review has some methodological strengths and limitations. To our knowledge, this is the first scoping review to identify narrative approaches in assessing care quality for youth and parents. A strength of our review is that we used input from patient/client organisations, regulators and academics to steer our focus. As we only reviewed English academic literature, we potentially miss non‐English studies and emerging approaches. We didn't assess methodological quality due to limited studies. This would be recommended when the repertoire of approaches expands in the coming years.

## 5 Conclusion

This review highlights various narrative approaches to gain a deep understanding of the diversity of youth and families using care, in addition to dominant professional perspectives. These approaches facilitate reflection, empowerment, and development of tailored services but also pose challenges regarding inclusivity, power dynamics, and creating impact. To address these, we suggest that researchers, policymakers, professionals and regulators in the field of care for youth and families reflect on how the narrative approaches they use could foster inclusivity, participation, and change.

## Author Contributions

**Eline Verheijen:** writing – original draft, formal analysis, methodology, investigation. **Suzanne Rutz:** writing – original draft, writing – review and editing, methodology, investigation. **Charlotte Barendregt:** writing – review and editing, investigation. **Anne Margriet Pot:** funding acquisition, writing – review and editing, supervision, project administration.

## Ethics Statement

The authors have nothing to report.

## Consent

The authors have nothing to report.

## Conflicts of Interest

The authors declare no conflicts of interest.

## Data Availability

The authors have nothing to report.

## Endnotes

1 We used the keywords listed in Table 1 for each database, adjusting the string as needed to run the specific database, but not adjusting the terms themselves.
